# Should We Continue Liver Transplantation in Spain for Hepatic Metastases from Neuroendocrine Tumors?

**DOI:** 10.3390/jcm15030938

**Published:** 2026-01-23

**Authors:** Andrea Boscà, Eva M. Montalvá, Marina Vila-Tura, Laura Lladó, Víctor López, Mikel Gastaca, Santiago Tomé, José M. Ramia, Javier Nuño, Fernando Rotellar, María Pérez, Óscar Caso, Mᵃ Mar Achalandabaso, Isabel Jaén, Carmen García, Pablo Ramírez, Rafael López-Andújar

**Affiliations:** 1 Department of Hepatobiliopancreatic Surgery and Transplantation, La Fe Health Research Institute (IIS La Fe), La Fe University Hospital, 46026 Valencia, Spain; 2 Centro de Investigación Biomédica en Red Enfermedades Hepáticas y Digestivas (CIBER-EHD), Health Institute Carlos III (ISCIII), 28029 Madrid, Spain; 3 Hepato-Biliary and Liver Transplantation Unit, Department of Surgery, Universitat de Barcelona, Hospital Universitari de Bellvitge, 08035 Barcelona, Spain; 4 Department of General, Visceral and Transplantation Surgery, Clinic and University Hospital Virgen de la Arrixaca, 30120 Murcia, Spain; 5 Hepatobiliary Surgery & Liver Transplantation Unit, Biocruces Bizkaia Health Research Institute, Hospital Universitario Cruces, University of the Basque Country, 48903 Bilbao, Spain; 6 Hepatology Unit, Internal Medicine Department, Hospital Universitario de Santiago, 15706 Santiago de Compostela, Spain; 7 Department of Surgery, Hospital General Universitario Dr. Balmis, 03010 Alicante, Spain; 8 Department of Liver Surgery, Instituto Ramon y Cajal de Investigacion Sanitaria, Hospital Universitario Ramon y Cajal, 28034 Madrid, Spain; 9 HPB & Liver Transplant Unit, Clínica Universidad de Navarra, 31008 Pamplona, Spain; 10 Institute of Health Research of Navarra (IdiSNA), 31008 Pamplona, Spain; 11 Department of Surgery, Hospital Regional de Málaga, 29010 Malaga, Spain; 12 Abdominal Organ Transplantation and General and Digestive Surgery Department, 12 de Octubre University Hospital, 28041 Madrid, Spain; 13 Department of General Surgery, Marqués de Valdecilla University Hospital-IDIVAL, School of Medicine, University of Cantabria, 39008 Santander, Spain; 14 Department of Surgery, Hospital Universitario de Badajoz, University of Extremadura, 06080 Badajoz, Spain; 15 HPB and Liver Transplant Unit, Department of General Surgery, University Hospital Central of Asturias (HUCA), University of Oviedo, 33011 Oviedo, Spain

**Keywords:** liver transplantation, liver metastases, neuroendocrine tumor

## Abstract

**Background/Objectives**: Despite the long-standing history of liver transplantation (LT) in Spain, no multicenter study has reviewed national outcomes for LT in metastatic neuroendocrine tumors (NETs). In the current era of transplant oncology, auditing these results is essential to refine patient selection and improve long-term outcomes. **Methods**: This retrospective observational study analyzed data from 13 centers, including 91 patients who underwent LT for NET between 1995 and 2024. Patients were stratified into two groups: Milan IN (those meeting the Milan criteria) and Milan OUT (the remainder). **Results**: Recurrence occurred in 57.1% of cases, and overall mortality was 51.6%. Of the 91 patients, 71 (78.0%) were Milan IN and 20 (22.0%) were Milan OUT. Five-year overall survival was 71.0% in Milan IN and 58.0% in Milan OUT, with a statistically significant difference. The 5-year disease-free survival (DFS) rate was 58.8% in Milan IN and 36.3% in Milan OUT; this difference was not statistically significant. **Conclusions**: In conclusion, strict adherence to Milan criteria and incorporation of modern prognostic factors are critical to optimize long-term survival in LT for NET. While the overall outcomes in this historical cohort are modest, future improvements are expected through more rigorous selection and the potential use of bridging or downstaging therapies.

## 1. Introduction

The prevalence of metastatic disease in gastroenteropancreatic neuroendocrine tumors (NETs) is very high, being seen in up to 75% of cases [1]. Despite their relatively slow and indolent growth, metastases represent a negative prognostic factor. Because they are primary tumors drained by the portal venous system, the liver is the main, and often only, organ involved in more than half of cases, followed by bone (12–20%) and lung (8–10%) [1,2]. Furthermore, some studies have reported a relatively predominant spread to the liver regardless of the primary tumor site [3].

Disease progression is often asymptomatic in nonfunctioning NETs, where the first manifestation may be liver metastases. By contrast, functioning tumors are usually diagnosed earlier, because they may be highly symptomatic and severely impair quality of life [4]. In advanced-stage NET patients, massive hepatic involvement often develops, leading to hepatic failure and subsequent death [2].

Radical resection remains the first-line treatment despite high recurrence rates within the first 2 years, and long-term cure is rarely achieved [5]. This suggests the presence of undetected microscopic hepatic disease not controlled before surgery, prompting some groups to advocate for liver transplantation (LT) in selected cases [6,7].

Since the first reports of LT for NET liver metastases (NET-LM), substantial changes have occurred as the tumor biology and prognostic factors have become better understood. Initial results of LT in NET-LM were discouraging, as it was often used as salvage therapy or last-resort treatment, with synchronous primary tumor resection and transplantation and suboptimal candidate selection. This led some multidisciplinary groups to reject LT altogether in these patients [8,9].

Currently, the most important guidelines for the management of NET-LM consider the therapeutic options: surgical resection in one or two stages (with or without locoregional treatments), palliative “debulking” surgery in patients with large symptomatic tumors, peptide receptor radionuclide therapy (PRRT), somatostatin analogs (SSA), and targeted molecular therapies. LT is recommended in < 1% of patients [5,10,11]. This low proportion is attributed to overall survival (OS) and disease-free survival (DFS) benefits only being obtained when applying the strict Milan selection criteria [2,12].

However, no randomized prospective trials have compared LT with other treatments such as surgical resection, systemic neoadjuvant therapies, or bridging strategies prior to transplantation, which could potentially broaden its indication in favor of patients [13,14].

This study evaluated the long-term outcomes of patients in OS and DFS who have undergone LT for NET-LM in Spain.

## 2. Materials and Methods

### 2.1. Study Design

This multicenter retrospective observational study involved 13 Spanish centers that include this indication in their LT protocols. Ninety-one patients underwent LT for NET-LM between January 1995 and August 2024. The study followed the STROBE guidelines for observational research.

The study was approved by the Ethics Committee of Hospital Universitari La Fe, Valencia (registry number 2024-0761-1) and sponsored by the Spanish Society of LT (SETH).

### 2.2. Data Collection

Data were collected anonymously, without patient identification. Participating centers linked patients’ clinical record numbers to an anonymous study identifier. Informed consent was waived given the absence of associated risk. All procedures were documented in accordance with Good Clinical Practice (GCP) guidelines.

### 2.3. Inclusion and Exclusion Criteria

Because this is a historical case series, all transplanted patients were included, even if they did not meet current accepted selection criteria for LT for NET-LM. Thus, unlike some series, patients transplanted with a primary tumor without portal drainage or with an unknown primary tumor were also included.

The patients included in the study had been deemed unqualified for surgical resection of liver metastases due to an inability to achieve R0 resection margins in all lesions or to guarantee sufficient future liver remnants. None of them had evidence of extrahepatic disease in CT images or somatostatin receptor scintigraphy (Octreoscan), and none presented with medical or surgical contraindications to LT. Exclusion criteria included the presence of extrahepatic disease in any imaging test or unresectable liver metastases from any origin other than NET.

### 2.4. Variables

The donor variables collected included age, sex, body mass index (BMI), cause of death, and type of donation. Recipient demographics included age, sex, BMI, comorbidities, histopathological features of the primary tumor, timing of metastasis diagnosis (synchronous or metachronous), tumor functionality, grade, lymph node involvement, surgical margin status, and Ki-67 index. Regarding hepatic involvement, the number of liver lesions, size of the largest lesion, and presence of vascular, lymphatic, or perineural invasion were recorded when available. Finally, postoperative complications, 90-day mortality, rejection, post-transplant tumor recurrence (DFS and site of recurrence), and treatment were documented. Immunosuppressive treatment was not included since it is a series that mixes historical and recent cases, and there is variability in the centers.

OS was defined as the interval between transplantation and death from any cause, censoring survivors at the date of last follow-up. DFS was defined as the interval between transplantation and tumor recurrence at any site, censoring patients at death or last recurrence-free follow-up. For survival analyses, patients were classified into two groups: those not meeting the Milan criteria, including tumors without portal drainage, portal-draining tumors without radical surgical resection, unknown primary tumors, G3 primaries, or metastatic burden > 50% of total liver volume (Milan OUT), and those fulfilling these criteria (Milan IN).

### 2.5. Statistical Methods

Continuous variables are expressed as mean ± standard deviation or median and interquartile range, depending on the data distribution (Shapiro–Wilk test). Categorical variables are described as counts and percentages. Survival curves were generated using the Kaplan–Meier method and compared to the log-rank test. All analyses were two-sided, with *p* < 0.05 considered statistically significant, and were performed using Stata software version 16.1 (StataCorp, College Station, TX, USA).

## 3. Results

**Figure 1:**
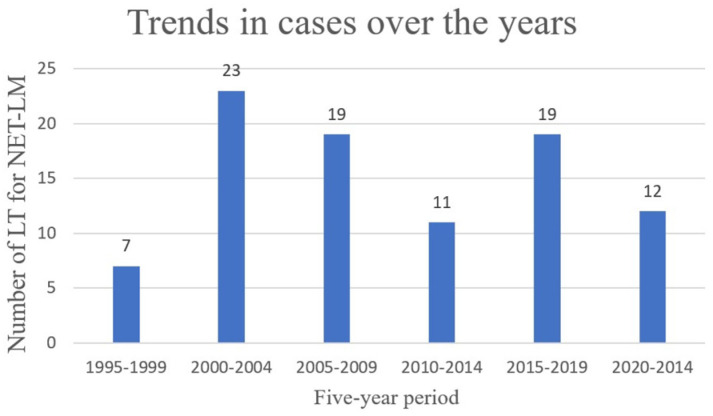
Trends in LT for NET-LM from 1995 to 2024.

In total, 91 patients underwent LT during the study period (Figure 1).

### 3.1. Population Characteristics

Donor and recipient age, sex, and BMI were comparable. Brain death accounted for the majority of organ donations (90%), with cerebrovascular accidents (CVAs) representing the leading cause of donor death, occurring in over 50% of cases (Table 1). Comorbidities were infrequent among recipients, reflecting their younger age compared to other transplant indications (Table 2).

### 3.2. Characteristics of the Tumor and Liver Metastases

The most frequent primary tumor site was the pancreas (54.9%), followed by the small intestine (26.4%). Hormone-secreting tumors accounted for only 30.8% of cases (Table 3).

Median time between diagnosis and primary tumor resection was 1.4 months (0.4–2.7). In more than 30% of cases, pathology reports following primary tumor resection were incomplete or insufficient. Median lymph node yield was 14 (7–19), with a median of three positive nodes (1–5). Tumor grade was G1 in 61.8%, G2 in 30.9%, and G3 in 7.3%. Ki-67 was available in 51 recipients: <3% in 19, 3–10% in 27, 11–20% in 4, and > 20% in 1 case. Of 87 patients with known primary tumors, margin status was reported in 75: 69 R0 (92%), 5 R1 (6.7%) and 1 case R2 (1.3%).

Hepatic metastases were synchronous in 66 cases (75.9%) and metachronous in 21 cases (24.1%), with a median interval of 34 months (16–48.7) between primary diagnosis and metastasis. Median time between primary tumor resection and transplantation was 18.8 months (10.3–52.4). Prior hepatic metastasectomy was attempted in 18 cases (19.8%). In over half of patients, hepatic lesions were innumerable (58.2%); in the remainder, a median of 16 lesions (9–17) were documented. Microscopic vascular invasion was present in 14.3% of cases, lymphatic invasion in 16.5%, and neural invasion in 4.4%, although it should be noted that these data were not available in approximately half of the pathology reports.

### 3.3. Post-Transplant Complications and Hospital Stay

Early post-transplant complications included 3 cases of primary non-function (3.3%), of which 2 underwent retransplantation and 1 died, and 11 hepatic artery thromboses (12.1%), 6 requiring retransplantation.

Late complications included 11 rejection episodes (12.1%), 10 managed with intensified immunosuppression, and 1 requiring retransplantation. Biliary complications were the most frequent late events (20.9%): there were 11 strictures, 7 fistulas, and 1 acute cholangitis.

Median Intensive Care Unit (ICU) stay was 3 days (2–5), and median hospitalization was 14 (10–20) days. Median follow-up was 6.5 years (1.7–13.4).

### 3.4. Recurrence

Recurrence occurred in 57.1% (52 patients). DFS was 84.9% at 1 year, 67.2% at 3 years, 53.5% at 5 years, and 28.7% at 10 years.

The most common recurrence pattern was involvement of multiple sites (32.7%), followed by lymph node metastases (28.6%) and bone lesions (19.2%). Systemic treatments for recurrence included SSAs in 80.8% of cases, targeted therapies such as everolimus or sirolimus in 38.5%, PRRT in 30.8%, and surgical resection in 17.3%. Surgical interventions targeted lymph node clusters, peritoneal implants, and solid organs including the pancreas, stomach, colon, spleen, and ovary. At the last follow-up, imaging studies revealed no pathological uptake in 3 cases (5.8%), disease stabilization in 16 patients (30.8%), and tumor progression in 33 cases (63.5%).

### 3.5. Mortality

**Figure 2:**
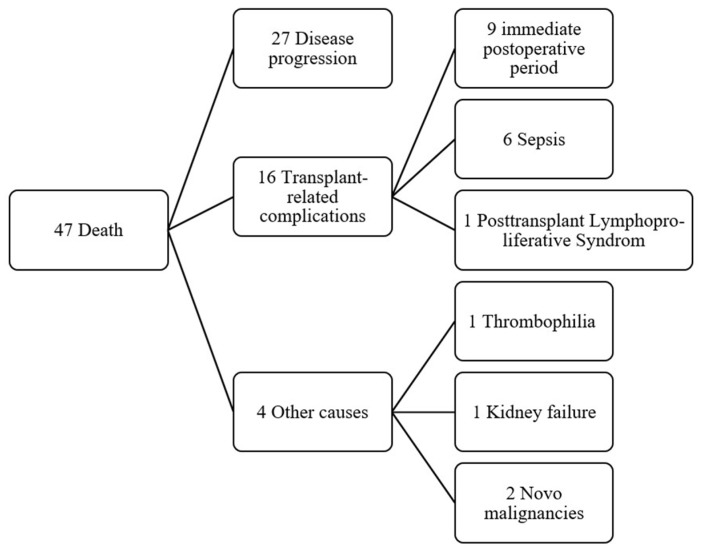
Flowchart with causes of death in transplant patients.

Overall mortality was 47 patients (51.6%). The observed OS rates were 85.6% at 1 year, 73.0% at 3 years, 68.1% at 5 years, and 49.4% at 10 years. Of the 47 deaths, 27 were attributed to disease progression and 20 to transplant-related complications (Figure 2). Notably, 9 patients (9.9%) died within 90 days post-transplant. Mortality was significantly higher among patients older than 60 years (84.6%, *p* = 0.005).

### 3.6. Milan IN vs. Milan OUT

**Figure 3:**
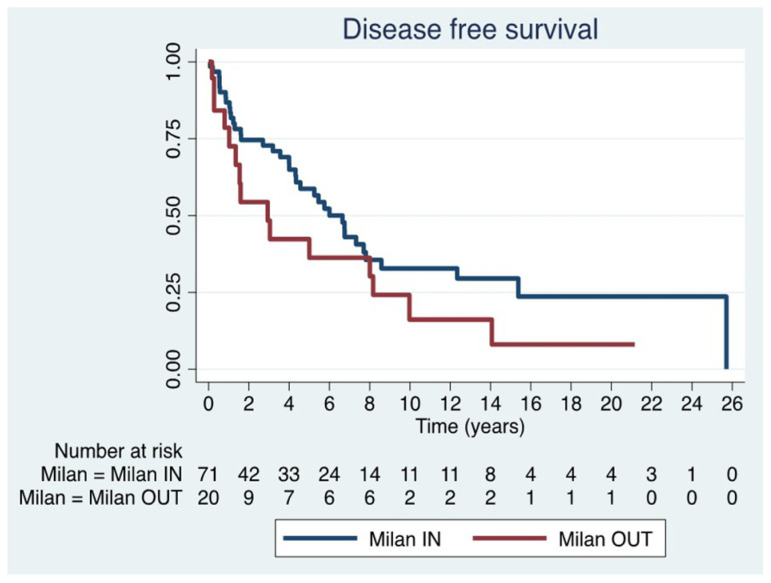
Disease free survival in Milan IN and Milan OUT.

Given the heterogeneity of this historical series regarding adherence to Milan criteria, a sub-analysis was performed in Milan IN (n = 71) and OUT (n = 20) patients. The median DFS was 3.6 years (IQR 0.9–7.3) in the former group and 1.6 years (IQR 0.6–8.1) in the latter group, with no statistically significant difference between the two groups (*p* = 0.11) (Figure 3) (Table 4).

Among post-transplant recurrences in Milan IN patients, disease stability was achieved in 37.8% (n = 14) and cure in 8.1% (n = 3); in Milan OUT patients, disease stabilization was achieved in only 13.3% (n = 2), with tumor progression occurring in the remainder (n = 13).

**Figure 4:**
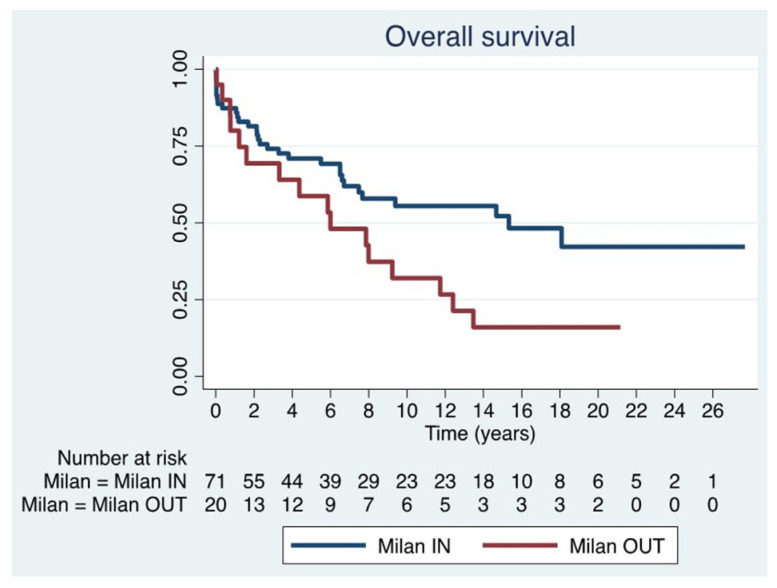
Overall survival in Milan IN and Milan OUT.

The median OS was 6.6 years (IQR 2.2–14.5) in Milan IN patients and 5.9 years (IQR 1.0–12.1) in Milan OUT patients, a statistically significant difference (*p* = 0.03) (Figure 4) (Table 5). Furthermore, analyzing only disease progression as a cause of mortality, it occurred in 45.2% of patients in the Milan IN group and 81.3% of those in the Milan OUT group.

## 4. Discussion

This study presents the first nationwide multicenter analysis of LT for NET in Spain, covering cases since the program’s inception in 1984. LT for NET remains a rare indication, accounting for < 1% of the more than 25,000 LTs performed in Spain since 1984 [15]. A substantial portion of this cohort (n = 41) underwent LT prior to the publication of the first NET-specific transplant criteria by Mazzaferro in 2007 [2].

In recent years, advances in tumor biology, improved imaging modalities, and the consolidation of LT as a standard procedure have significantly influenced patient selection. The introduction of the Milan criteria in 2007 marked a turning point [2]. Most centers now follow these or similar guidelines, such as ENETS (Ki-67 < 10%) (11) or the 2025 UNOS criteria, which extend eligibility to tumors with Ki-67 < 20% [16]. In parallel, recent international guidelines and reviews have highlighted the increasing relevance of precision medicine, targeted therapies, and emerging tools such as liquid biopsy in the management of neuroendocrine neoplasms, particularly in advanced disease settings [17]. While these strategies were not available for most patients included in our historical cohort, they may become increasingly relevant in future transplant oncology approaches.

Surgical resection remains the preferred treatment for resectable NET liver metastases with curative intent [7]. However, a long-term cure is uncommon, with recurrence rates of 70–90% within 2 years, even after R0 resections [5,7,13]. Cytoreductive surgery, while not equivalent to radical surgery or LT, has shown survival benefits when > 70% of the tumor burden is removed [18,19].

Accurate pre-transplant staging is critical. Triple-phase CT, contrast-enhanced MRI, and, increasingly, 68Ga-DOTATATE PET (specificity up to 98%) are used to rule out extrahepatic disease [20,21]. In our series, this modality was rarely available; Octreoscan was predominantly used, although it is now considered less effective [22].

Radical resection of the primary tumor is essential prior to LT. The 2013 European Liver Transplant Registry study emphasized that LT should not be performed concurrently with primary tumor resection [8]. In our cohort, some patients underwent LT without adequate resection or with an unknown primary tumor, lacking key prognostic data such as tumor grade and Ki-67 index [7]. These limitations compromise prognosis and render the cohort unrepresentative of current transplant candidate selection standards.

LT timing is another critical factor. The Milan criteria recommend demonstrating disease stability for at least 6 months prior to LT, during which point transplantation offers the greatest benefit [23,24]. Patients with progression during this interval should be excluded from LT and considered for alternative therapies. In our cohort, the median time to LT was 18.8 months (range: 10.3–52.4), with considerable variability.

No prospective randomized trials have compared LT with other medical treatments [1]. In Caplin et al. in 2014, SSAs improved 5-year progression-free survival (PFS: ~40%) versus placebo, although OS remained unchanged [25]. These results are not directly comparable to DFS outcomes post-LT, as many patients lacked primary tumor resection. Other therapies, including everolimus, sirolimus, and especially PRRT, have gained prominence in advanced disease [26,27]. Consequently, some centers have ceased offering LT for NET, and this indication is no longer considered in half of the transplant centers in Spain. Kunz et al. recently reported that, despite the 2013 North American Neuroendocrine Tumor Society (NANETS) consensus on the survival benefits of surgery [28], only one-third of eligible patients undergo surgical treatment, likely influenced by the increasing use of systemic therapies over the past decade [29]. The expansion of systemic treatments has not spurred research into bridging or downstaging strategies for LT or surgery [30], but rather has led to a focus on their standalone efficacy in improving OS and PFS [25]. A German clinical trial (NCT01201096) is currently investigating PRRT prior to LT in selected cases. In our series, only one patient meeting the Milan criteria received PRRT pre-LT, with no recurrence after 3.8 years of follow-up.

Perioperative mortality (9.9%) in our series is comparable to historical data (8). Advanced age (> 60 years) was associated with significantly worse outcomes, reinforcing the need for younger candidates with limited comorbidities.

The reported incidence of hepatic artery thrombosis (12.1%) reflects the actual rate observed in this nationwide cohort. We were unable to identify a single explanatory factor, as neither donor nor recipient age was unusually high, and no consistent technical or perioperative risk factors could be identified retrospectively across centers. It is also possible that the underlying oncologic nature of the disease, which is known to be associated with a prothrombotic tendency, may have contributed to this finding. In addition, a proportion of patients who developed hepatic artery thrombosis had previously undergone liver surgery for hepatic resections, which may have increased technical complexity at the time of transplantation and could also have played a contributory role. Given the historical nature of part of the cohort and the well-known evolution of surgical techniques and perioperative management over the study period, this observation should therefore be interpreted with caution.

Several retrospective studies have reported outcomes in LT for NET, often without distinguishing Milan IN and OUT patients. Systematic reviews cite 5-year OS of 47–70% and recurrence rates of 31.3–56.8%, reflecting heterogeneous cohorts [30]. Le Treut in 2013 identified three unfavorable prognostic factors (hepatomegaly, concurrent primary tumor resection, and age > 45 years), reporting a 5-year OS of 52% and a DFS of 30%. Patients with ≤1 negative factor achieved an OS of 79% and a DFS of 57% [8]. The first Milan IN series was published by Mazzaferro in 2016 and reported a 5-year OS of 97.2% and a DFS of 86.9% [12]. In a 2022 follow-up with additional transplanted patients, the same researcher reported an OS of 95.5% and a DFS of 75% [23] (Table 6). More recent large-scale retrospective studies include Valvi et al. (UNOS registry, n > 200), which reported a 5-year OS of 64.9% and a DFS of 43.9%, with comparisons to HCC and cholangiocarcinoma showing no significant differences [31].

Eshmuminov et al. in 2023 reported a 5-year OS of 75% and a DFS of 64.2% in Milan IN patients [7]. Our results align with these series, although we did not replicate the outcomes of Mazzaferro’s Milan IN cohort [12]. In our data, OS was superior in Milan IN patients, with a sustained benefit over time. While DFS did not significantly differ between Milan IN and OUT patients, recurrence in the latter group was more aggressive and accounted for > 80% of mortality.

This study had some limitations, including a lack of pathology data, historical variability in patient selection, and inconsistent classifications, which may have led to underestimations of the differences between the Milan IN and OUT groups. This may have favored the null hypothesis and produced conservative DFS and OS estimates. Additionally, early patients were treated > 20 years ago, when transplant teams lacked current experience, potentially inflating morbidity and mortality.

Nevertheless, our findings support strict adherence to selection criteria, including comprehensive staging with 68Ga-DOTATATE PET, radical primary tumor resection, known Ki-67 and grade, and a period of disease stability prior to LT [35,36]. Optimal patient selection by a multidisciplinary team that includes oncologists, endocrinologists, hepatologists, transplant surgeons, pathologists, radiologists, and nuclear medicine specialists is essential [37,38]. Rigorous adherence to the Milan criteria and modern prognostic factors is critical to achieving long-term survival comparable to other transplant indications. While overall outcomes in this historical series are modest, future results will likely improve in patients meeting the Milan criteria, especially with the integration of systemic therapies as neoadjuvant strategies.

## Figures and Tables

**Table 1:** General characteristics of donors.

	Donors
Age (years)	48.9 +/− 17.7
Sex	59.3% Men
	40.7% Women
BMI *	25.4 +/− 3.9
Types of donations	90.1% DBD
	8.8% DCD
	1.1% Living donation
	58.2% Stroke
Cause of death	27.5% TBI
	6.6% Cerebral anoxia
	7.7% Other causes

* BMI: Body Mass Index; DBD: Donation after Brain Death donors; DCD: Donation after circulatory donor; TBI: Traumatic Brain Injury.

**Table 2:** General characteristics of recipients.

	Recipients
Age (years)	48.8 +/− 11.2
Sex	63.7% Men
	36.3% Women
BMI *	25.3 +/− 4.1
	19.8% Hypertension
	16.5% Diabetes
Comorbidities	9.4% Dyslipidemia
	16.7% Smoking
	6.7% Alcohol consumption

* BMI: Body Mass Index.

**Table 3:** Location of the primary tumor and functionality.

Primary Tumor	Nonfunctioning Tumors	Hormone-Secreting Tumors	
Pancreas = 50		8 Insulin	2 Gastrin
	33	2 Somatostatin	2 Glucagon
		2 Serotonin	1 VIP *
Small intestine n = 24	20	4 Serotonin	
Lung n = 5	-	5 Serotonin	
Colon-rectum n = 5	5	-	
Unknown n = 4	3	1 Serotonin	
Duodenum n = 2	1	1 Serotonin	
Appendix n = 1	1	-	

* VIP: Vasoactive intestinal polypeptide.

**Table 4:** One, 5-, 10- and 15-year DFS * rates in both groups: Milan IN and Milan OUT.

	DFS * 1 Year	DFS * 5 Year	DFS * 10 Year	DFS * 15 Year
Milan IN	86.8% (IC 75.4–93.2)	58.8% (IC 44.4–70.5)	32.8% (IC 19.8–46.6)	29.6% (IC 16.8–43.7)
Milan OUT	78.6% (IC 52.5–91.4)	36.3% (IC 15.1–58.1)	16.1% (IC 3.2–38.2)	8.1% (IC 0.6–29.3)

* DFS: Disease Free Survival.

**Table 5:** One, 5-, 10- and 15-year OS * rates in both groups: Milan IN and Milan OUT.

	1-Year OS *	5-Year OS *	10-Year OS *	15-Year OS
Milan IN	87.3% (IC 62.0–93.2)	71.0% (IC 58.6–80.2)	55.5% (IC 41.9–67.2)	52.2% (IC 38.0–64.7)
Milan OUT	80.0% (IC 55.1–91.9)	58.7% (IC 34.0–76.8)	32.0% (IC13.2–52.7)	16.0% (IC 4.0–35.3)

* OS: Overall Survival.

**Table 6:** Results of the papers with the highest volume in LT for NET.

Year	Author	n	Milan Criteria	5-Year OS	5-Year DFS
2011	Nguyen [32]	184	No	49.2%	-
2013	Le Treut [8]	213	No	52%	30%
2015	Nobel [33]	230	No	63%	-
2015	Sher [34]	85	No	52%	-
2016	Mazzaferro [12]	42	Yes	97.2%	86.9%
2020	Valvi [31]	206	No	64.9%	43.9%
2022	Maspero [23]	48	Yes	95.5%	75%
2023	Eshmuminov [7]	225	No	73%	64.2%
2025	Spanish group	91 71	No Yes	68.1% 71%	53.5% 58.8%

## Data Availability

Data are available under reasonable request and after the approval of the corresponding author in order to protect the privacy of our patients.

## Abbreviations

The following abbreviations are used in this manuscript:

LT	Liver transplantation
NET	Neuroendocrine Tumors
NET-LM	NET liver metastases
PRRT	Peptide receptor radionuclide therapy
SSA	Somatostatin analogs
OS	Overall Survival
DFS	Disease-free survival
GCP	Good Clinical Practice
CT	Computed tomography
BMI	Body mass index
CVAs	Cerebrovascular accidents
DBD	Donation after Brain Death donors
DCD	Donation after circulatory donor
TBI	Traumatic Brain Injury
VIP	Vasoactive intestinal polypeptide
ICU	Intensive Care Unit
MRI	Magnetic resonance imaging
PFS	Progression-free survival
